# Utilization of peripheral nerve feedback at a preconscious level

**DOI:** 10.3389/fnins.2024.1336431

**Published:** 2024-03-14

**Authors:** Nabeel Hasan Chowdhury, Dustin James Tyler

**Affiliations:** ^1^Department of Biomedical Engineering, Case Western Reserve University, Cleveland, OH, United States; ^2^Louis Stokes Cleveland VA Medical Center, United States Department of Veterans Affairs, Cleveland, OH, United States

**Keywords:** peripheral nerve stimulation, dorsal column, sensorimotor interaction, neuroaxis, preconscious, simple reaction time, Piéron’s law, attentional blink

## Abstract

**Introduction:**

Sensorimotor integration is important, if not required, when using our hands. The integration of the tactile and motor systems is disrupted in individuals with upper limb amputations because their connection to their fingertips is lost. Direct cortical stimulation allows for modality and location matched perceptions; however, studies show that the time to process and act upon direct cortical feedback significantly exceeds the time to do the same with naturally produced tactile feedback. Direct cortical stimulation does not engage multiple parallel structures in the brain stem meant to integrate tactile feedback with signals from the motor system at a sub-perceptual or pre-perceptual level before the somatosensory cortex is involved. While reasonable to assume, it is not known if the artificially generated signals will engage the same peripheral tactile pathways to the pre-perceptual and perceptual structures as natural tactile sensation. Our hypothesis is that pre-perceptual structures will process the electrically generated neural activity as it would naturally generated neural activity.

**Methods:**

In this study, stimulation of the median nerve in multiple subjects’ residual limbs produced modal, and location matched sensory perceptions in their hands. We found the time to process different stimuli using simple reaction time tests in three different formats.

**Results:**

We showed the minimum time to process peripheral nerve stimulation and initiate a motor plan is similar to naturally generated tactile feedback and is processed upwards of 50 – 175 ms faster than visual feedback alone. We also found the effect of stimulation intensity on the rate of feedback processing follows the same trend of natural sensory feedback, Piéron’s law indicating that the unimodal processing of PNS is similar to natural touch. Finally, we found that tactile feedback given to a pre-perceptual level is again used in the motor plan.

**Discussion:**

Taken together, we conclude that peripheral nerve stimulation engages the pre-perceptual pathways of the brain, and hence demonstrate advantages of peripheral restoration of sensory inputs.

## Introduction

Most upper limb amputees rely on visual feedback when using a myoelectric prosthesis. Reliance on vision restricts myoelectric prosthesis users to focusing on the intricacies of singular tasks, resulting in slow, error prone performance along with mental and physical fatigue (Chadwell et al., 2018). The restoration of haptic feedback reduces the physical and mental energy required to use a myoelectric prosthesis (Witteveen et al., 2012). One of the simplest methods of haptic feedback restoration is sensory substitution by vibration motors in the prosthesis socket (Witteveen et al., 2012; Riet et al., 2013). When vibrotactile feedback is used as an error signal, able bodied individuals had an improved speed to correct for errors during the task and reduced the effort in completing a task or multiple tasks (Sklar and Sarter, 1999; Riet et al., 2013; Rosenbaum-Chou et al., 2016). However, for these able-bodied individuals, the feedback could be given directly to their fingertips, which is not possible for amputees. Feedback given on the residual limb is less intuitive and shows slower reaction times when used in grasping tasks (Jansen et al., 2004). Further difficulties arise when multiple vibrations represent complex grip patterns. Creating a mental mapping of multiple vibrations on the forearm to different grasp patterns becomes confusing, especially when also combing vibration intensities to convey graded, differential force information (Antfolk et al., 2013).

Producing tactile sensations perceived with visually matched mode and location on the hand reduces this perceptual mapping problem. These sensations often occur in those whose sensory neurons have grown into their skin or were moved to the skin using targeted sensory restoration (Hebert et al., 2014). Touching the reinnervated skin will produce the perception that the hand is being touched in the locations those neurons used to innervate and is understood much more intuitively (Hebert et al., 2014). Direct cortical stimulation of the somatosensory cortex also produces the perception of touch on the hand. However, while feedback was felt in functionally relevant locations, direct stimulation of the cortex processes feedback 25–100% *slower* than the reaction time to visual feedback alone (Odoherty, 2009; Godlove et al., 2014; Caldwell et al., 2019). Furthermore, in tests utilizing nonhuman primates, if the animal made an error or failed to complete a trial, reaction times slowed further on subsequent trials (Bensmaia, 2015). Increasing the perceived intensity of stimulation by increasing the level of injected current to the cortex is known to decrease tactile feedback processing time (Sombeck and Miller, 2019). However, requiring a high intensity stimulus for useful tactile information affects the ability to perceive minor grip changes and adjustments. Given intensity matched cortical and vibrotactile feedback, vibrotactile feedback is still perceived faster (Christie et al., 2021; Dexheimer et al., 2022).

There are two leading theories to describe slower time in processing tactile perceptual feedback from cortical stimulation. The first is that direct stimulation of the cortex equally activates excitatory and inhibitory neurons, requiring a reinterpretation of the stimulation after it has been provided (Odoherty, 2009; Godlove et al., 2014; Caldwell et al., 2019). The second is that cortical stimulation bypasses several structures along the ascending pathway to the somatosensory cortex that normally perform pre-perceptual processing of the tactile stimuli in route to the cortex (Odoherty, 2009; Godlove et al., 2014; Bensmaia, 2015; Caldwell et al., 2019). Without stimuli engaging the multiple preprocessing centers along this pathway, all processing is offloaded onto the cortex, which requires an increase in perceptual effort, and hence, delays the time from processing to motor action.

Multiple studies have suggested there are important subcortical areas of the brain that help preprocess tactile feedback as it ascends from the periphery to the cortex. Firstly, Libet showed that peripheral stimuli as short as a single action potential produce prolonged activity in the somatosensory cortex suggesting there is an amplification of the signal along the path to the cortex (Libet, 1965). He also found that threshold tactile feedback in the periphery is perceived earlier than equivalent cortical feedback even when cortical stimulation is given hundreds of milliseconds earlier (Libet et al., 1979; Libet, 1993). This phenomenon is also consistent with Christie’s work which show that touch, whether generated naturally or by peripheral nerve stimulation, delayed by 111 ± 62 ms is still perceived as simultaneous with visual feedback (Christie et al., 2019). Libet suggested that there is an extralemniscal pathway separate from the typical tactile pathway that generates this conscious referral to an earlier time of perception that is not accessible when stimulating the cortex directly.

More recent studies show that perception of tactile sensation originates from thalamocortical and cortico-cortical interactions between multiple areas across the cortex even to areas typically occupied by other senses (Imanaka et al., 2002; Dijkerman and Haan, 2007; Henschke et al., 2014; Redinbaugh et al., 2019). There also exists a separate pathway to the cortex for tactile feedback that produces no perception. This pathway originates in the medial region of the posterior nuclei of the thalamus (POM) and sends tactile feedback to the posterior parietal cortex (Dijkerman and Haan, 2007; Redinbaugh et al., 2019). This pathway bypasses the ventroposterior lateral (VPL) nucleus of the thalamus, the main input to the somatosensory cortex (Redinbaugh et al., 2019). Individuals with a lesion in the VPL nucleus and an intact POM nucleus had no perception of tactile sensation, but still were able to point to regions on the hand where they were touched (Dijkerman and Haan, 2007; Redinbaugh et al., 2019). Without a need for tactile perception, this indicates that tactile feedback has a link to creating a motor plan through the posterior parietal cortex.

**Figure 1:**
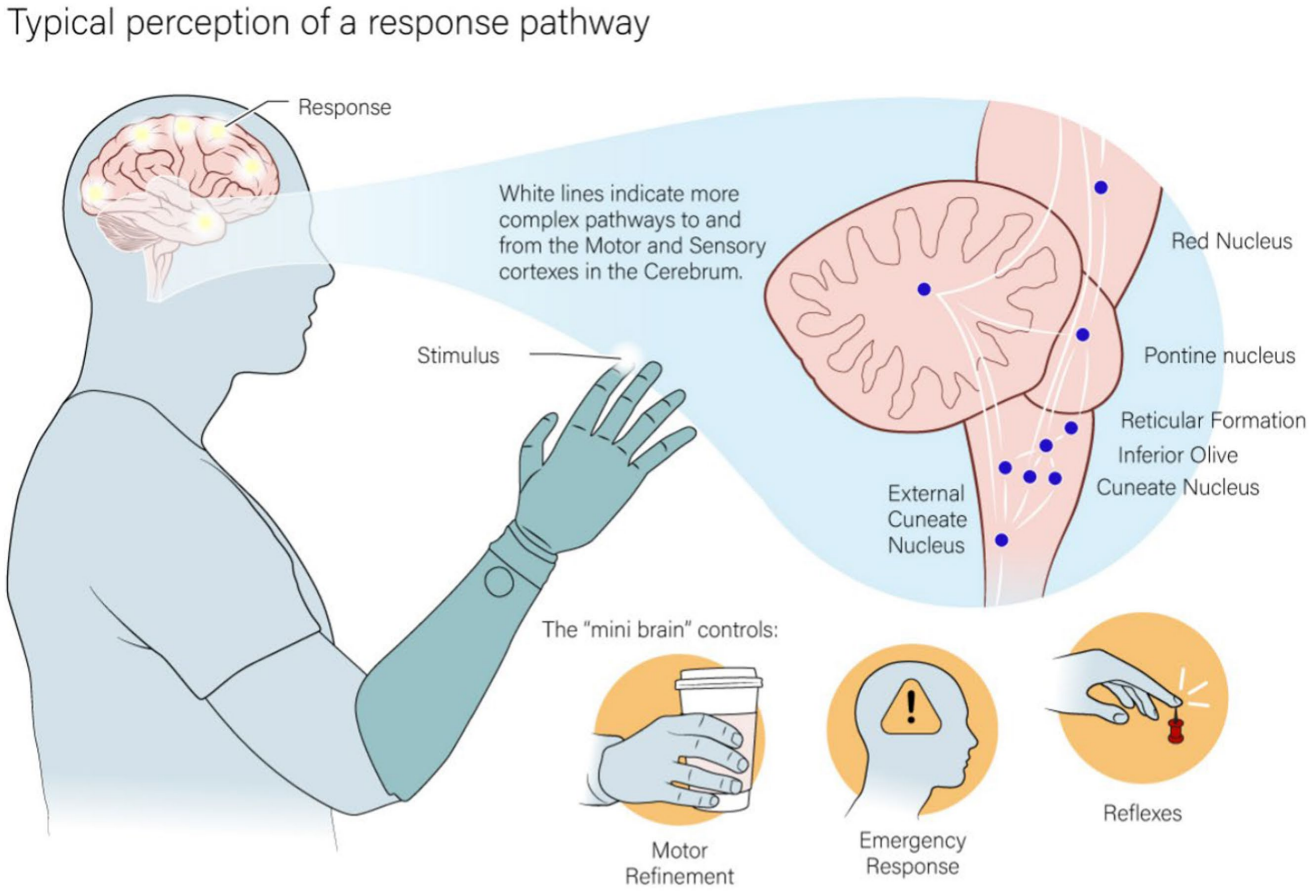
The pathway that both natural and artificial tactile feedback ascends through interact with a dense parallel pathway of nuclei in the lower parts of the brain. This area is used in the base processing of tactile feedback and to react to simpler motor action or actions that require quick responses.

The earliest synapse in the ascending tactile pathway, the cuneate dorsal column nuclei, also has extensive branching to multiple subcortical regions (Figure 1). Each of these secondary nuclei have an important role in more basic sensorimotor functions and motor corrections that do not require conscious control - one of the most important of which is the cerebellum (Loutit and Potas, 2020). Stimulation past the brainstem will bypass the typical inputs to the cerebellum, essentially skipping one of the most important areas in comparing tactile and motor activity.

Natural tactile feedback interacts with the brain at many different levels along the ascending pathway from the periphery to the cortex. Peripheral nerve stimulation will connect through the pathways of natural tactile input, including the same subcortical and cortical structures. It is expected that the signal is preprocessed by the same structures that process natural tactile feedback (Libet, 1965, 1993; Libet et al., 1979; Imanaka et al., 2002; Odoherty, 2009; Godlove et al., 2014; Bensmaia, 2015; Caldwell et al., 2019). It is not known, however, if the artificial firing patterns will result in equivalent processing and response as natural tactile feedback. The pre-perceptual usage of tactile feedback in these subcortical structures is likely essential for integration with the rest of the sensorimotor system (Stein et al., 1976; Wallace et al., 1996; Mac Lean, 2000; Riet et al., 2013; Redinbaugh et al., 2019; Loutit and Potas, 2020; McFadyen et al., 2020). Starting earlier in the tactile pathway will likely require a much less refined signal to be useful. To this end, we can utilize the full pathway from the periphery by stimulating peripheral nerves.

Here, we present three experiments to test our hypothesis that artificially generated peripheral nerve stimuli are processed with similar characteristic to naturally generated tactile stimuli. Firstly, a simple reaction time test evaluated the processing time of peripheral nerve stimulation compared to naturally generated visual and vibrotactile feedback. The second test measured the relationship between intensity of peripheral nerve stimulation and time to process tactile feedback. Previous studies show that Piéron’s law describes how the time to process multiple forms of naturally generated sensation decreases with increases in intensity (Pins and Bonnet, 1996; Imanaka et al., 2002; Van Maanen et al., 2012; Loutit and Potas, 2020). A close fit to Piéron’s law would imply that intensity changes to peripheral nerve stimulation map well to naturally generated sensation. Finally, we tested the pre-perceptual execution of a preplanned motor action by weak peripheral nerve stimuli masked from perception during a backmasking experiment. Triggering the motor plan by a masked peripheral nerve stimulation examines the interaction between artificially generated tactile feedback and pre-perceptual tactile pathways.

## Methods

### Subjects

Three male human volunteers with unilateral, upper limb loss (one right arm and two left arm) participated in this study. Subject 1 has a right trans-radial amputation due to a traumatic injury in 2004 and was implanted with three 8-contact flat interface nerve electrodes (FINEs) (Tyler and Durand, 2002) around his median, ulnar, and radial nerves in 2012. Subject 2 has a left trans-radial amputation due to a traumatic injury in 2013 and was implanted with two 16-contact CFINEs (Dweiri et al., 2016; Freeberg et al., 2017) around his median and ulnar nerves in 2016. Subject 3 has a left trans-radial amputation due to a traumatic injury in 2012 and was implanted with two 16-contact CFINEs around his median and ulnar nerves in 2017. Subjects 2 and 3 also have implanted EMG electrode pairs in eight of their forearm muscles. For all tests, only the median nerve cuff was used. Each subject came in for a test session between 1 and 2 h every 2–6 weeks, depending on their availability. Each test was performed sequentially in the order presented here. The full study occurred over 2 years and subjects came in as their personal obligations fluctuated over time. This resulted in some subject being available more or less for some experiments, or not at all in the example of subject 2 in the second experiment. In total, subject 1 participated in 6 experimental sessions of the simple reaction time test, 3 sessions of the intensity-based reaction time test, and 9 sessions of the final experiment with backmasking. Subject two participated in 5 sessions of the simple reaction time test and 3 sessions of the final experiment with backmasking. Subject 3 participated in 11 sessions of the simple reaction time test, 6 sessions of the intensity-based reaction time test, and 17 sessions of the final experiment involving backmasking.

Of note are the significant number of tests run over the course of several years. The effect of learning in this test could have skewed the simple reaction times for each subject over time if learning occurred. However, Baker shows that performing a simple reaction time with a set of subjects periodically over the course of a year did not result in noticeable learning or effect on reactions times (Baker et al., 1986). We see the same when looking at our subjects’ simple reaction time results over time (Supplementary Figure 1) as all subject stayed consistent or slightly go slower over time. Only in one case tit the subject seem to get faster over time. Therefore, in all experiments, an average reaction time represents the basic processing time.

All study devices and procedures were reviewed and governed by an U.S. Food and Drug Administration Investigational Device Exemption, the Cleveland Department of Veterans Affairs Medical Center Institutional Review Board, and the Department of the Navy Human Research Protection Program. Informed consent was obtained from all subjects.

### Electrical stimuli

Trains of charge-balanced, square, bi-phasic, current-controlled, cathode first stimulation pulses were delivered to individual contacts on the electrodes. The contacts were chosen so that the perceived locations in response to stimulation were on the thumb in all tests as well as a contact that selectively produced independent perceptions on the index finger in the intensity-based reaction time test. The pulse amplitude and pulse frequency of the stimuli were constant, and the pulse width was manipulated to change the perceived intensity. When finding perceived intensity before each experiment, the pulse width of the stimulus is set to 250 μs, the pulse frequency is set to 100 Hz, and the pulse amplitude is raised in 10 μA steps until the intensity reaches the level that the subject would use when grasping an object firmly. This level is defined as “comfortable. Next, the pulse width is dropped to the lowest possible value where the subject felt the stimulus 100% of the time. This level is defined as threshold. In the intensity-based reaction time experiment, we substituted “comfortable” with “max comfortable.” When finding “max comfortable” the pulse width is increased further in the first step until just before the subject is no longer comfortable using a higher intensity or the pulse width before any muscle contractions were elicited, whichever is lower. Pulse amplitudes were set at the beginning of the experiment to give at least a 100 μs difference in the pulse widths between max comfortable and threshold intensities.

The projected field locations of touch perception are on the tip of the thumb in all subjects for all tests with the addition of the tip of the index finger in the intensity-based reaction time test. We chose these locations because these percepts have been reliably activated over the full time each subject has had their implant and these two locations are important during grasping. Changes in intensity result in growing of these locations in the thumb and index finger, but no movement of the location across threshold to max comfortable intensities.

### Simple reaction time test

**Figure 2:**
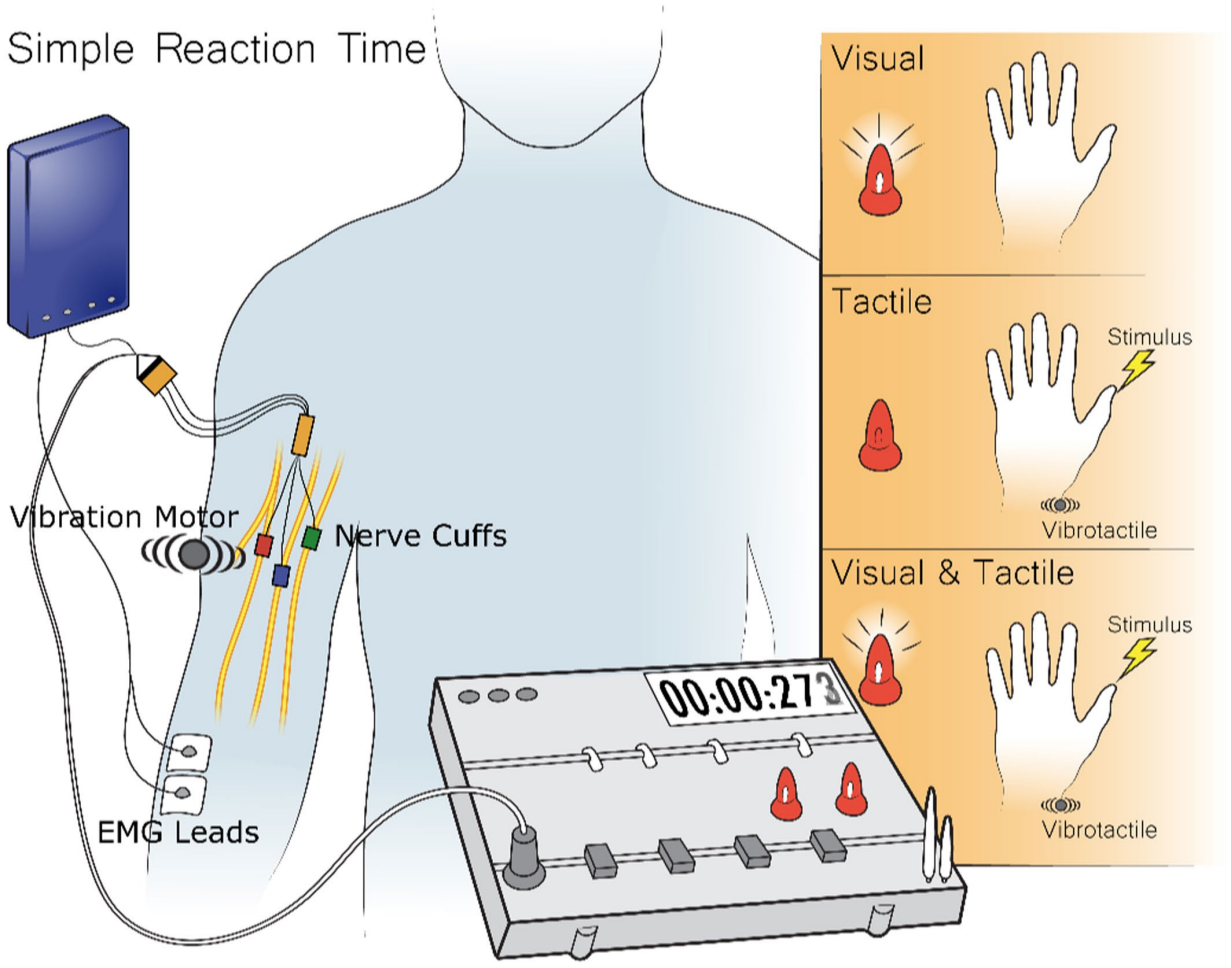
During the Simple Reaction Time Test the subject receives three different stimuli types: visual, tactile, and visual-tactile. Visual stimuli come from two LEDs in front of the subject, tactile stimuli come either from stimuli to implanted electrodes or a vibrotactile stimulus on the skin about the electrodes, and visual-tactile feedback comes from the combination of the previous two stimuli. The reaction time to the stimulus is recorded by the time to record a muscle contraction which is either recorded from the surface or through implanted EMG electrodes.

One of the most basic measures of the minimal time to process and respond to a sensory stimulus is the simple reaction time test (SRT) (Libet et al., 1979; Taylor and Mccloskey, 1990; Craig and Evans, 1995; Pins and Bonnet, 1996; Forster et al., 2002; Imanaka et al., 2002; Miller and Ulrich, 2003; Jansen et al., 2004; Van Maanen et al., 2012; Godlove et al., 2014; Woods et al., 2015; Caldwell et al., 2019; Loutit and Potas, 2020). In a simple reaction time test the subject performs a simple action, like pressing a button as soon as they perceive any sensation. Sensations are applied at random times. Because the response action is known beforehand, the subject preplans their motor action before the stimulus arrives. The delay between when the feedback arrives and when the action is completed represents the time to perceive the stimulus, process it, and then trigger their motor plan (Stein et al., 1976; Godlove et al., 2014). To examine peripheral nerve stimulation, the simple reaction time test measured visual feedback, tactile feedback from either peripheral nerve stimulation or vibrotactile stimulation, and the combination of visual feedback with either tactile feedback modality (Figure 2). The simple action requested of the subject was to contract their forearm muscles as soon as they felt or saw any of the feedback methods. This EMG signal was recorded either through surface EMG electrodes for Subject 1 or implanted EMG electrodes for Subjects 2 and 3.

Visual stimuli were presented through one of two LEDs directly in front of the subject. Tactile feedback was either provided through stimuli sent to the FINE cuff around the subject’s median nerve or to a vibration motor placed on the skin above the location of the FINE electrode. This placement intended to keep roughly the same signal length of the nerve for the vibration motor to the brain as the cuff stimulation to the brain. Visual-tactile feedback was given through the pairing of simultaneous visual stimuli from the LEDs with tactile stimuli from either the vibration motor or the FINE cuff. Before each trial, both LEDs illuminated for 3 s and then extinguished. Stimuli occurred after a random interval of 4–7 s. The test consisted of a randomized set of fifty visual, fifty tactile, and fifty visual-tactile trials divided between three blocks with a break as long as the subject desired in between each block. Tactile feedback in each overall test came from either the vibration motor or the FINE cuff, but not both. Each experimental day consisted of equal numbers of tests with peripheral nerve stimulation and vibrotactile stimulation. This resulted in twice as many visual feedback trials as tactile or visual-tactile trials from each tactile modality as the visual trails were repeated in trials with the two tactile feedback modalities.

The subject was told to perform a voluntary muscle contraction as soon as they perceived any of the stimuli. For Subjects 2 and 3, reaction time was recorded from implanted EMG electrodes, and for Subject 1, this was recorded from surface electrodes placed over the sites the subject uses for control of his myoelectric device. The reaction time was measured as the inflection point from the baseline toward the first major peak in the filtered, rectified, and smoothed EMG signal.

Data for all subjects is normally distributed and we performed an ANOVA analysis with a Tukey correction to find the difference between the groupings for visual feedback, peripheral nerve stimulation, and the combination of visual feedback and peripheral nerve stimulation. A data summary is in Table 1.

**Table 1:** The reaction times for different modalities of sensory feedback.

	Subject 1			Subject 2			Subject 3		
	*n*	μ (ms)	95% CI (ms)	*n*	μ (ms)	95% CI (ms)	n	μ (ms)	95% CI (ms)
Visual	252	328.76	315.39, 342.13	186	275.00	261.00, 288.90	213	233.42	222.87, 243.98
Stimulation	133	277.30	258.90, 295.70	60	99.07	74.45, 123.68	125	179.55	165.78, 193.33
Visual + Stim	125	188.38	169.39, 207.36	74	74.00	80.53, 124.86	124	154.63	140.80, 168.46
Vibrotactile	125	194.61	175.63, 213.60	96	142.85	123.39, 162.31	92	164.78	148.72, 180.83
Visual + Vibrotactile	124	178.92	159.86, 197.98	93	126.80	107.03, 146.57	95	155.82	140.02, 171.62

*n*, number of trials; μ, mean reaction time; CI, −/+ values of the 95% confidence interval.

### Intensity based reaction time

While the simple reaction time test finds the minimum time to process a basic stimulus, higher intensity stimuli can still shorten the time to process the stimulus. All above-described experiments were performed with stimulation intensities at a subject defined level for “comfortable.” This level of stimulation is the level they would use at home was the maximum stimulus level used for this test. Previous literature on the effect of increasing intensities of natural visual, auditory, gustatory, olfactory, and tactile feedback show the shortening of the simple reaction times to those stimuli follow the same trend, a modified power curve called Piéron’s law (Pins and Bonnet, 1996; Imanaka et al., 2002; Van Maanen et al., 2012; Loutit and Potas, 2020). If artificial tactile feedback from peripheral nerve stimulation is processed by the brain in a similar way to naturally generated stimuli, the effect of increasing peripheral nerve stimulation intensity should also follow Piéron’s law (Eq. 1).


(1) $$SRT=\beta I^{-\alpha }$$

**SRT** is the mean reaction time at each intensity value, β represents the size of the spread in reaction times above the minimum reaction time, I is the intensity of the stimulus, and α represents an exponential decay specific to each sensory modality. The I term is typically a quantitatively measurable value such as lumens or decibels for visual or auditory feedback, respectively. The intensity of peripheral nerve stimulation is traditionally determined by the subject’s reported experience of the stimulation parameters, but these values can shift on a day to day or even hour by hour basis for the same stimulation parameters. A more objective measure of intensity is the activation charge rate (ACR) (Graczyk et al., 2016). This is essentially the difference between charge of the cathodic pulses in the stimuli at the test level minus the charge at threshold multiplied by the pulse frequency. The charge of the pulse is the pulse amplitude multiplied by the pulse width. In this experiment, the intensity was modulated by keeping a constant pulse amplitude and pulse frequency while changing the pulse width. Since pulse amplitude and pulse frequency are constant, they are common factors of the ACR equation and are incorporated in the beta term (Eq. 2). The range of pulse widths were from the 100% threshold of perception to the maximum comfortable intensity before muscle contractions occur.


(2) $$I\approx ACR=PF\times PA\left(PW-PW_{\mathrm{thresh}}\right)=k\left(PW-PW_{\mathrm{thresh}}\right)$$

Substituting Equation 2 into Equation 1 produces the following equation:


(3) $$SRT=\beta {\left(PW-PW_{\mathrm{thresh}}\right)}^{-\alpha }$$

Where k is incorporated into β (Equation 3). The experimental intensities were divided into 10 equally spaced pulse width values in the range between threshold and maximum comfort for a stimulation channel on the thumb and index finger. These were found for each stimulation channel at the start of the experiment and fixed for the duration. The 20 stimuli were randomized for intensity and if the stimuli were felt on the thumb or index fingertip. The two LEDs used in the previous experiment again prompted the subject that the next trial was coming after a random delay of 1–3 s. Reaction times were also recorded by the same EMG measure as in the simple reaction time test.

The test involved five blocks of one hundred trials per block, with five trials per intensity level. The one hundred trials were presented randomly. The subject was again told to contract their forearm muscles as soon as they perceived any tactile feedback. Subject 3 performed this test 6 times resulting in 150 samples per intensity level. The fit for the equation was determined in Python using a least squares fit. This experiment only involved Subject 1 and Subject 3, as Subject 2 was unavailable to run the full test multiple times.

### Measuring the pre-perceptual tactile connections to the motor plan

The previously described experiments measure simple processing, but do not provide insight into the underlying neural circuits engaged in the reaction time. Natural touch is used both above and below the level of perception. Full integration of our peripheral nerve stimulation with the motor plan requires integration at a pre-perceptual level. This experiment investigated the integration of pre-perceptive processing with artificial peripheral nerve stimulation. Backwards masking has been shown to suppress the *perception* of weak tactile stimuli while still demonstrating their engagement in pre-perceptual usage (Bachmann, 1984; Taylor and Mccloskey, 1990; Imanaka et al., 2002). Forwards and backwards masking take advantage of the attentional blink phenomenon to supply feedback at a pre-perceptual level. The main difference between a sub-perceptual and a pre-perceptual stimulus is, while a sub-perceptual stimulus can never be felt, a pre-perceptual stimulus can be if given enough attention. An attentional blink test uses a strong stimulus to take the focus away from a weak, pre-perceptual stimulus. This works if the strong stimulus is presented before or after the weak stimulus if the time between them is small (Libet, 1965, 1993; Libet et al., 1979; Bachmann, 1984; Taylor and Mccloskey, 1990; Craig and Evans, 1995; Imanaka et al., 2002; Nieuwenstein et al., 2009; Dehaene and Changeux, 2011). Backwards masking is a common test used to analyze how a pre-perceptual visual or tactile stimulus can trigger faster motor responses when paired with a perceived stimulus (Bachmann, 1984; Taylor and Mccloskey, 1990; Imanaka et al., 2002). The purpose of this test is to determine if pre-perceptual processing of stimuli is still possible with an artificial stimulus from peripheral nerve stimulation.

**Figure 3:**
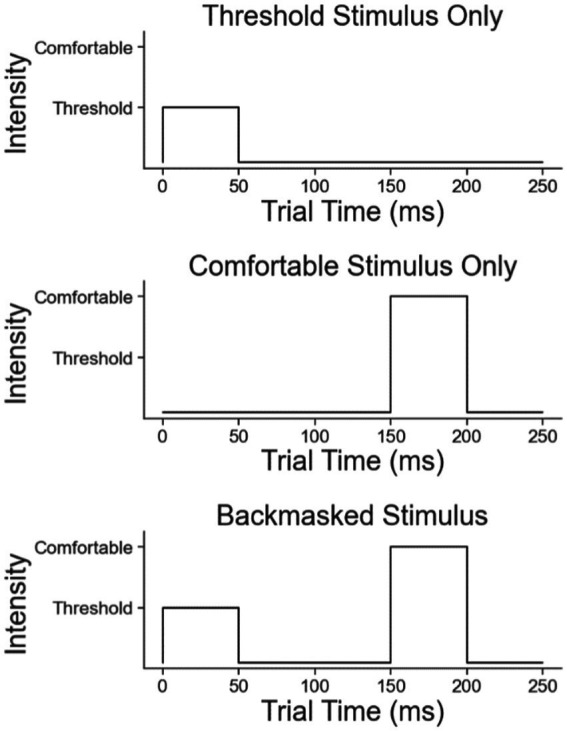
The three trial types in the backmasking experiment: the weak stimulus, the strong stimulus, and the backmasking stimulus.

There were three different types of stimulation paradigms (Figure 3). The first paradigm was a weak stimulus burst for 50 ms, but strong enough to be perceived 100% of the time if applied as a single pulse burst. The second paradigm was a single strong stimulus burst given at the subjects’ specified “comfortable” intensity level for 50 ms and delayed by 150 ms from when the test started. The third paradigm was the combination of the first two stimuli, with a weak stimulus followed by the strong one with a 100 ms interburst interval. This third case is the backmasking trial in which the strong stimulus masks the weak stimulus.

The subjects were asked to react as fast as possible with a muscle contraction to any stimuli felt. After reacting they identified how many bursts they felt. Reaction times were measured from the start of the first stimulus the subjects indicated they felt. If the subjects felt one stimulus in the masked case, the reaction time was measured from the stronger stimulus start because it is unlikely they only felt the weak stimulus and not the strong one in this case. The subjects were told that the number they reported was less important than the speed of reaction. If the subjects felt one burst in the backmasking trial, the strong stimulus masked the weak stimulus, and if they felt two pulses, no masking occurred. Subjective measures of masking like this one correlate closely with EEG recordings confirming masking (Dehaene and Changeux, 2011). To ensure the subjects did not feel both stimuli together as one long stimulus, catch trials were run interspersed with the regular trials. In the catch trials, two strong stimuli were presented with the same timing as the backmasking trials. If the subjects report two pulses with the catch trial and only one with the backward masking, masking was confirmed.

Data for Subject 1 and 3 is normally distributed and we performed a t-test to detect if there was a difference between the reaction time to the backmasked and strong stimulus. Subject 2 did not have normally distributed data, so we use the non-parametric Mann–Whitney test between the same two groups. A summary of the data in this test is in Table 2.

**Table 2:** The statistical properties of the data from the backmasking trial for the three subjects involved.

	Subject 1			Subject 2			Subject 3		
	*n*	μ (ms)	95% CI (ms)	*n*	μ (ms)	σ (ms)	*n*	μ (ms)	95% CI (ms)
Weak stimulus	328	691.17	674.70, 707.64	56	359.70	172.70	516	604.08	592.32, 615.83
Strong stimulus	430	595.94	581.56, 610.32	66	378.50	120.20	679	512.23	501.99, 522.48
Backmasked stimulus	172	573.60	550.90, 596.40	42	249.81	58.17	443	476.16	463.48, 488.84

### EMG filtering

**Figure 4:**
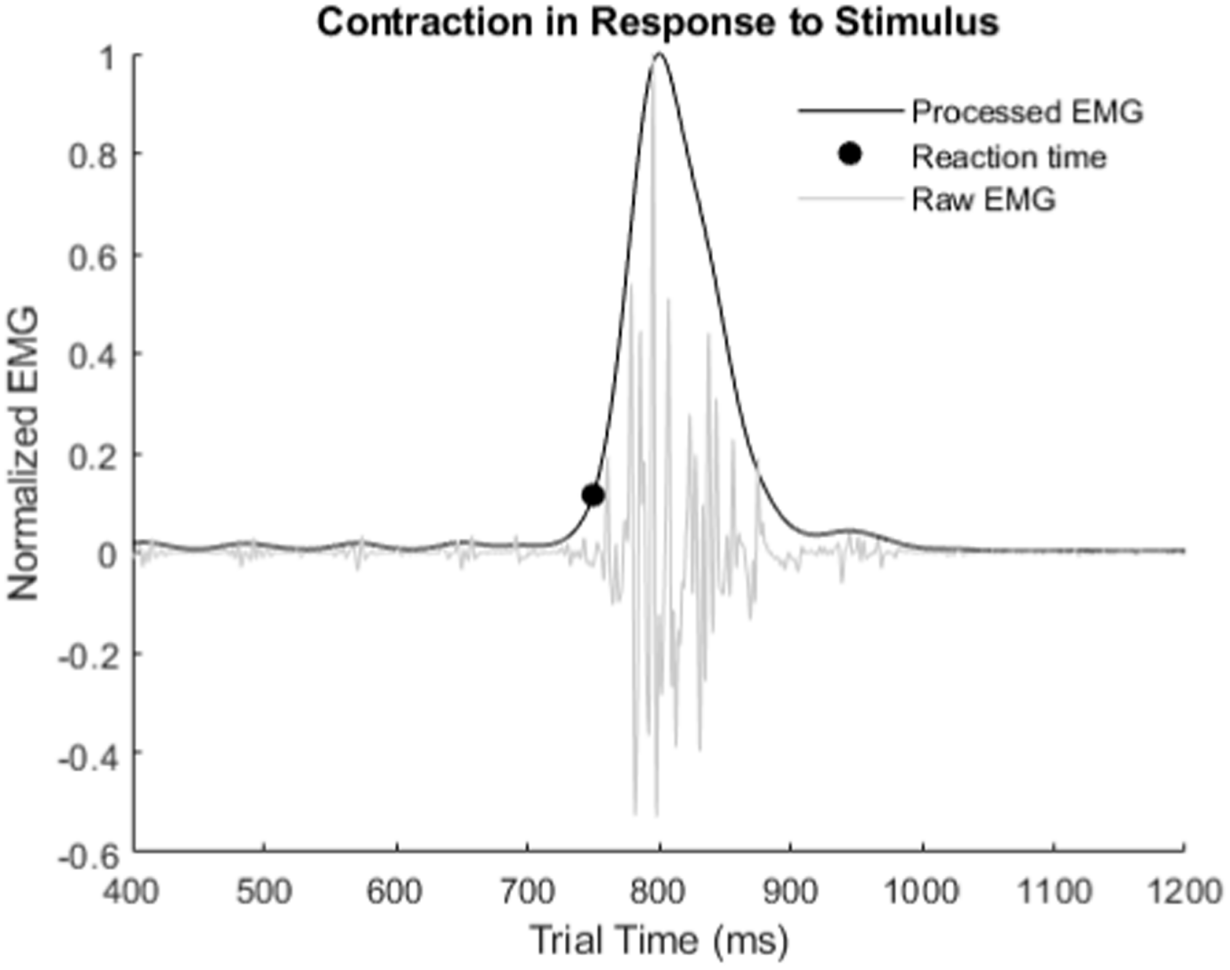
Example of how reaction times were measured from recorded EMG. Reaction time is from stimulus onset to the inflection point of the major rise in the EMG signal.

All three experiments required the recording of EMG signals from the residual forearm of the subject to measure reaction time (Figure 4). The first stage filter for each EMG channel was a 500 Hz 4th order IIR lowpass filter with Butterworth filter characteristic in the Ripple Grapevine™ stimulator (Ripple, LCC, Salt Lake City, UT). The next stage was a full wave rectification and normalization by the maximum measured EMG in the trial. The final stage was an averaging then smoothing of all EMG channels during each trial with a 100th order 0.5 Hz Low Pass FIR filter to find the major peak in the contraction. This final filter processes the signal forwards and backwards to prevent a phase shift in the reaction time. The reaction time was the first point 10% above baseline of the major rise in the EMG contraction relative to the start of the stimulus onset.

### Timing circuit

Relying on the internal computer time would result in a high amount of variability in recorded reaction times (Woods et al., 2015). The Ripple Grapevine™ stimulator, which supplied the peripheral nerve stimulation, also has analog and digital input/output. To time when visual and/or tactile stimuli were applied, the Grapevine set a synchronous digital signal to the high state. When the stimuli ended, the Grapevine™ reset the signal back to the low state. The EMG recording through the Grapevine used the same clock as the digital signal. The timing of the digital signal indicated the test start and stop times. We segmented out the EMG signals during each trial based on these starting and stopping times. A second digital signal activated the vibrotactile motor. The vibrotactile motor had a measured delay of between 1 and 2 ms from the time the synchronization timing pulse was sent to when the vibrotactile motor vibrated. The characterization of delay between the digital signal and peripheral nerve stimuli was about 0.5 ms. Since reaction times to stimuli were on the order of hundreds of milliseconds, these small delays do not affect the results.

## Results

### Simple reaction time

**Figure 5:**
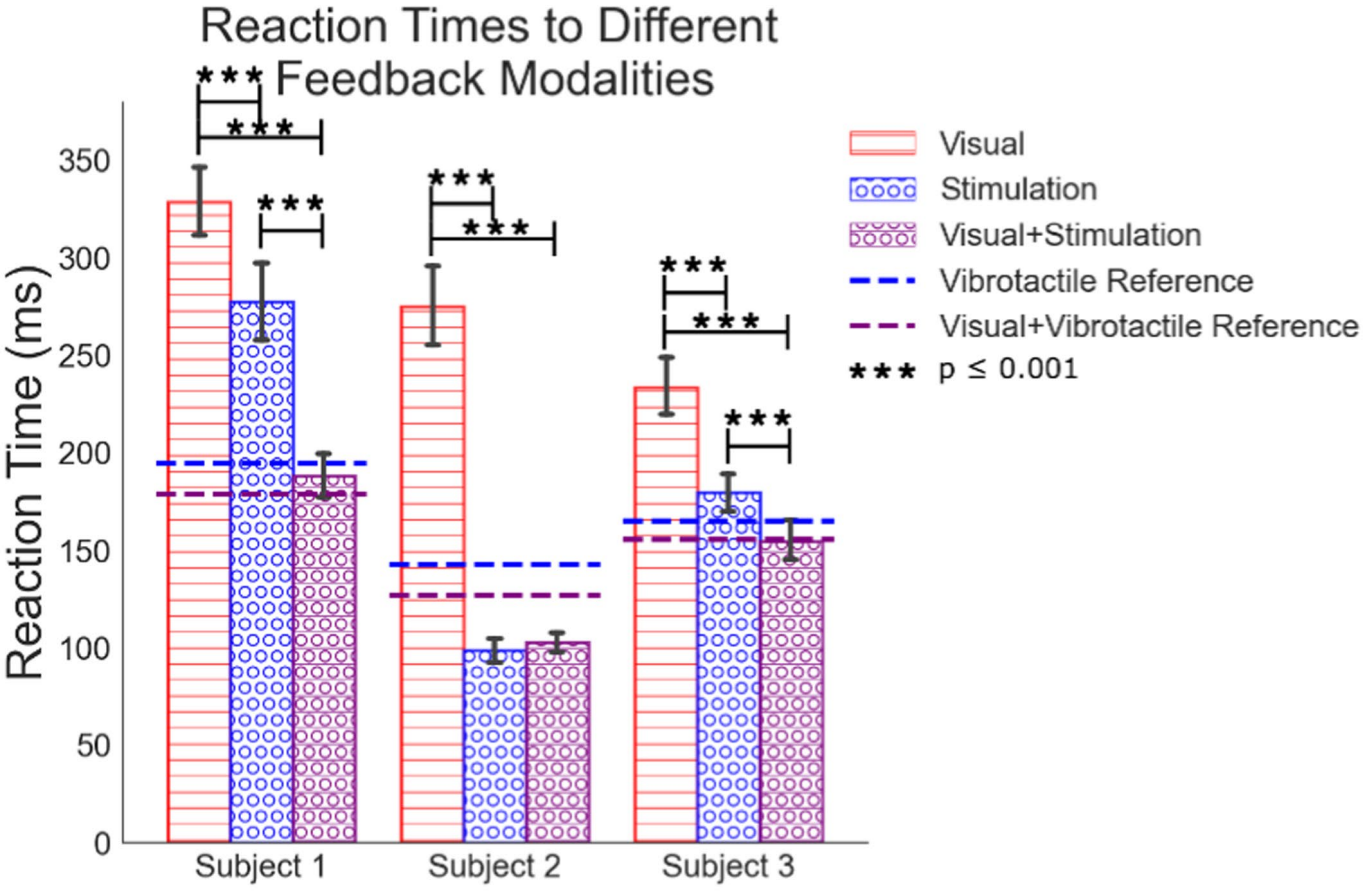
Mean simple reaction times to visual feedback, peripheral nerve stimulation, the combination of the visual feedback with the peripheral nerve feedback. Reference lines for how subjects react to vibrotactile feedback are included.

The average reaction times due to each feedback modality are significantly different for several of the five modalities examined (Table 1; Figure 5). The analysis excludes times in the source data below 50 ms and above 1,000 ms as outliers. They represent times corresponding to guessing when to react or from missing the cue to react, respectively.

All sets of data are normally distributed. We used an ANOVA analysis with a Tukey comparison to test for differences between processing feedback modalities presented to each subject (Figure 5). Peripheral nerve feedback always results in faster reaction times compared to visual feedback alone. Subject 1’s peripheral nerve feedback alone results in slower reaction times compared to the vibrotactile feedback alone, but still faster than visual alone. In verbal feedback from this subject, they stated that it was “confusing” to react to a tactile sensation on his hand that was not correlated with an actual visual cue of something physically touching his hand. This may slow his time to react. For all subjects, the combination of visual feedback with peripheral nerve stimulation results in reaction times faster than vision alone. Combined vision with peripheral feedback is not statistically different than peripheral feedback alone except for subject one once he had the LED turning on as a change in the environment, he could reference the combined feedback to. In all cases except subject 2, peripheral stimulation with vision reaction times trends faster than peripheral feedback alone. This suggests the processing of combined peripheral nerve stimulation and visual feedback results in some integration of the senses, but it is not clear if it is full multisensory integration. Subject 1 also seemed to get faster at reacting to the combination of visual and stimulated feedback (Supplementary Figure 1) which may indicate an increase in confidence or understanding of the electrical stimuli over time when paired with visual feedback.

**Figure 6:**
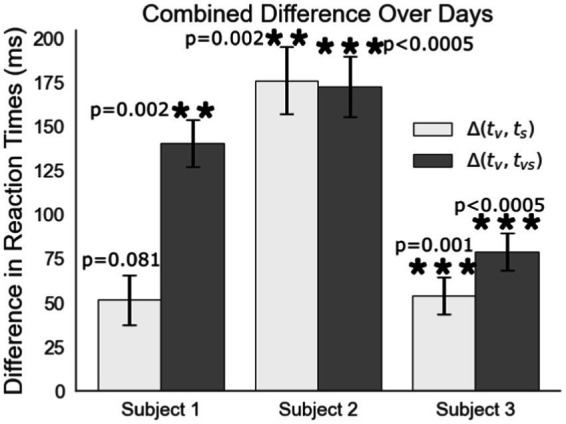
Difference comparison of the reaction to visual feedback (t_v_) to the reaction time to peripheral nerve stimulation (t_s_) or visual feedback combined with peripheral nerve stimulation (t_vs_).

Visual feedback response times are up to 175 ms slower than responses any feedback involving artificial tactile feedback (Figure 6). Each difference in response time is between experiments on the same experimental day with 5, 6, and 11 days of trials for subjects 1, 2, and 3, respectively. Shown is the average of the differences from all days. Finding the reaction time differences on each experimental day individually mitigates day to day drift in reaction times due to subjective intensity differences in the feedback between experimental days. These results represent a shorter processing time of peripheral nerve feedback compared to visual feedback alone. For Subjects 2 and 3, peripheral nerve stimulation is significantly faster than visual feedback and it is nearly significant for Subject 1 despite the subject’s comments about their cognitive dissonance with getting sensation that did not correspond to a visual touch on the hand. For all subjects, the addition of stimulation to the visual feedback greatly shortens the processing time to respond.

### Intensity based reaction time

**Figure 7:**
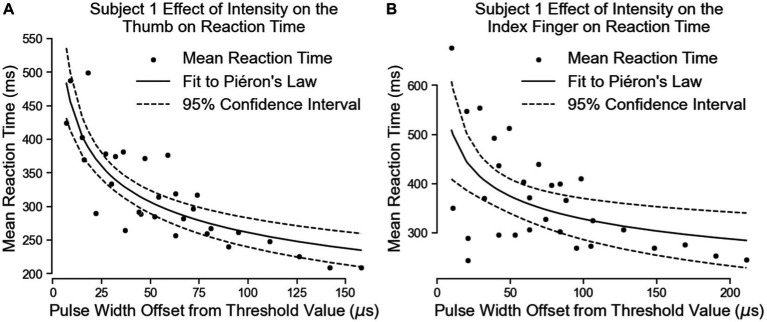
The effect of intensity on Subject 1’s simple reaction times. The results for the thumb are shown in panel **(A)** and the results for the index finger are in panel **(B)**. Left on the figure is closer to threshold and right is closer to a “comfortable” level of stimulus.

Subject 1’s data of reaction times fit Piéron’s Law for both the thumb (Figure 7A; Table 3) and index fingers (Figure 7B; Table 3). The parameters of the equation for the thumb are β = 759.8 and α = 0.2319. The root mean squared error is 41.5 ms. The parameters for the index finger are β = 788.0 and α = 0.1902. The root mean squared error is 86.92 ms. The magnitude of Subject 1’s thumb reaction times toward the right of Figure 7A are of note here as these reaction times had the same intensity as the simple reaction times, but in this case, Subject 1 preferred to keep his eyes closed to eliminate any visual distractions.

**Table 3:** Fit of each stimulation type to Piéron’s Law.

		*α*	*β*	RMSE
Subject 1	Thumb	0.2319	759.8	41.5 ms
	Index	0.1902	788.0	86.92 ms
	Both	0.1942	729.1	75.78 ms
Subject 3	Thumb	0.1841	675.5	37.11 ms
	Index	0.1887	691.0	52.15 ms
	Both	0.1871	685.5	45.27 ms

The first two rows show the fit of each location individually and the third row shows the fit of the two locations with their data pooled.

**Figure 8:**
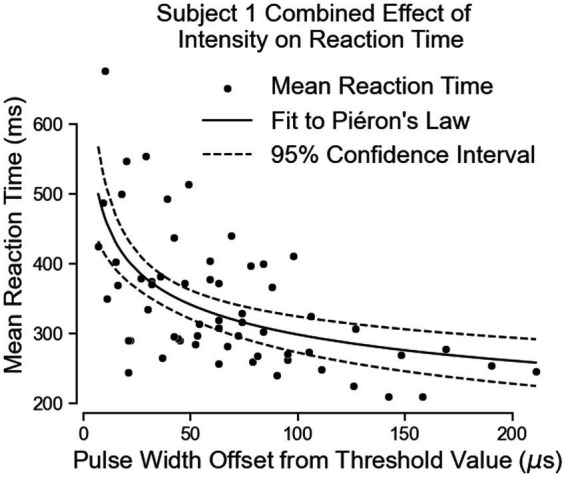
The combination of all of Subject 1’s data together follows Pieron’s Law for the intensity to stimulus processing time.

Both equations show similar fit parameters despite the subject reporting different intensities. This suggests that the model is agnostic to the location of sensation. When the data of both locations are combined and the curve fit redone, the variables are once again very similar to the previous results (Figure 8; Table 3). The fit equation has a β value of 729.1 and an α value of 0.1942 with a root mean squared error of 75.78 ms, which is similar to the individual fit errors.

**Figure 9:**
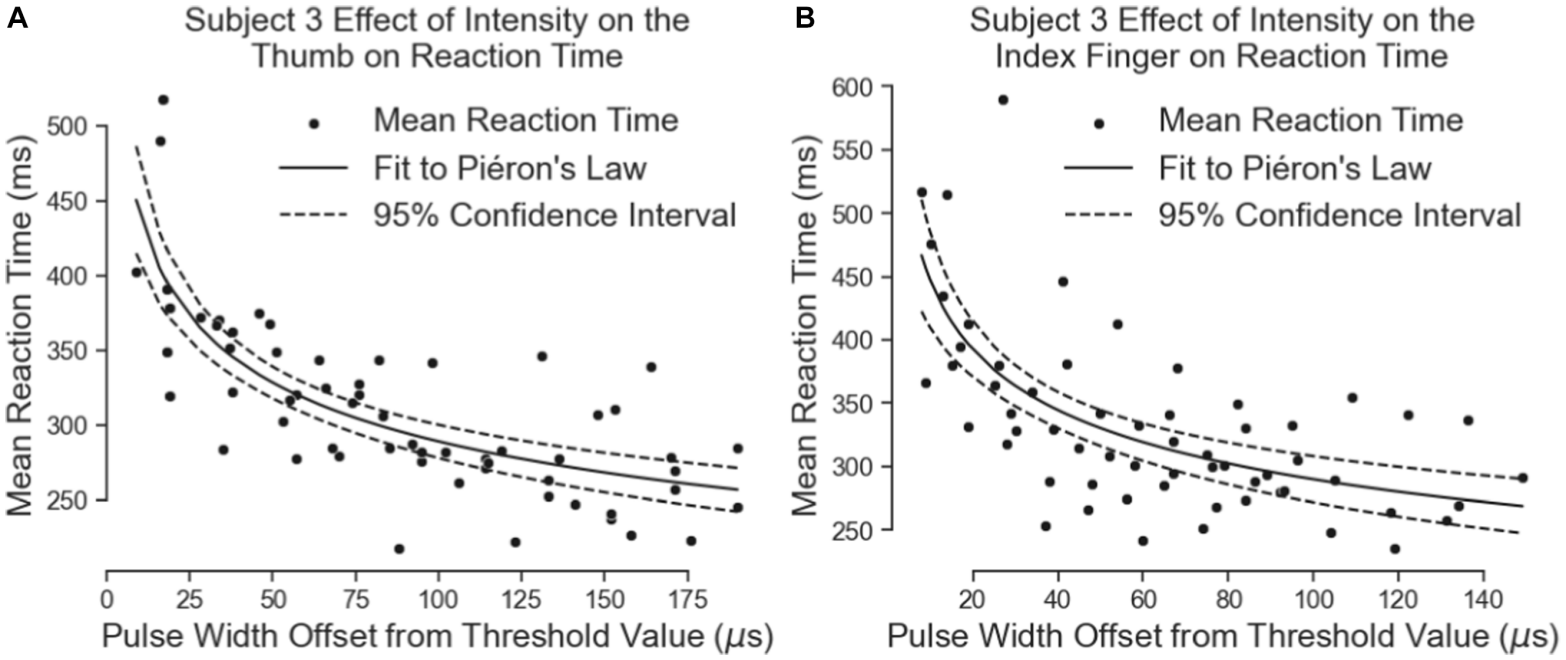
The effect of intensity on simple reaction times. The results for the thumb are shown in Panel **(A)** and the results for the index finger are in Panel **(B)**.

Subject 3’s data of reaction times fit Piéron’s Law as well for both the thumb (Figure 9A; Table 3) and index fingers (Figure 9B; Table 3). The parameters of the equation for the thumb are β = 675.5 and α = 0.1841 with a root mean squared error of 37.11 ms. The parameters for the index finger are β = 691.0 and α = 0.1887 with a root mean squared error of 52.15 ms.

**Figure 10:**
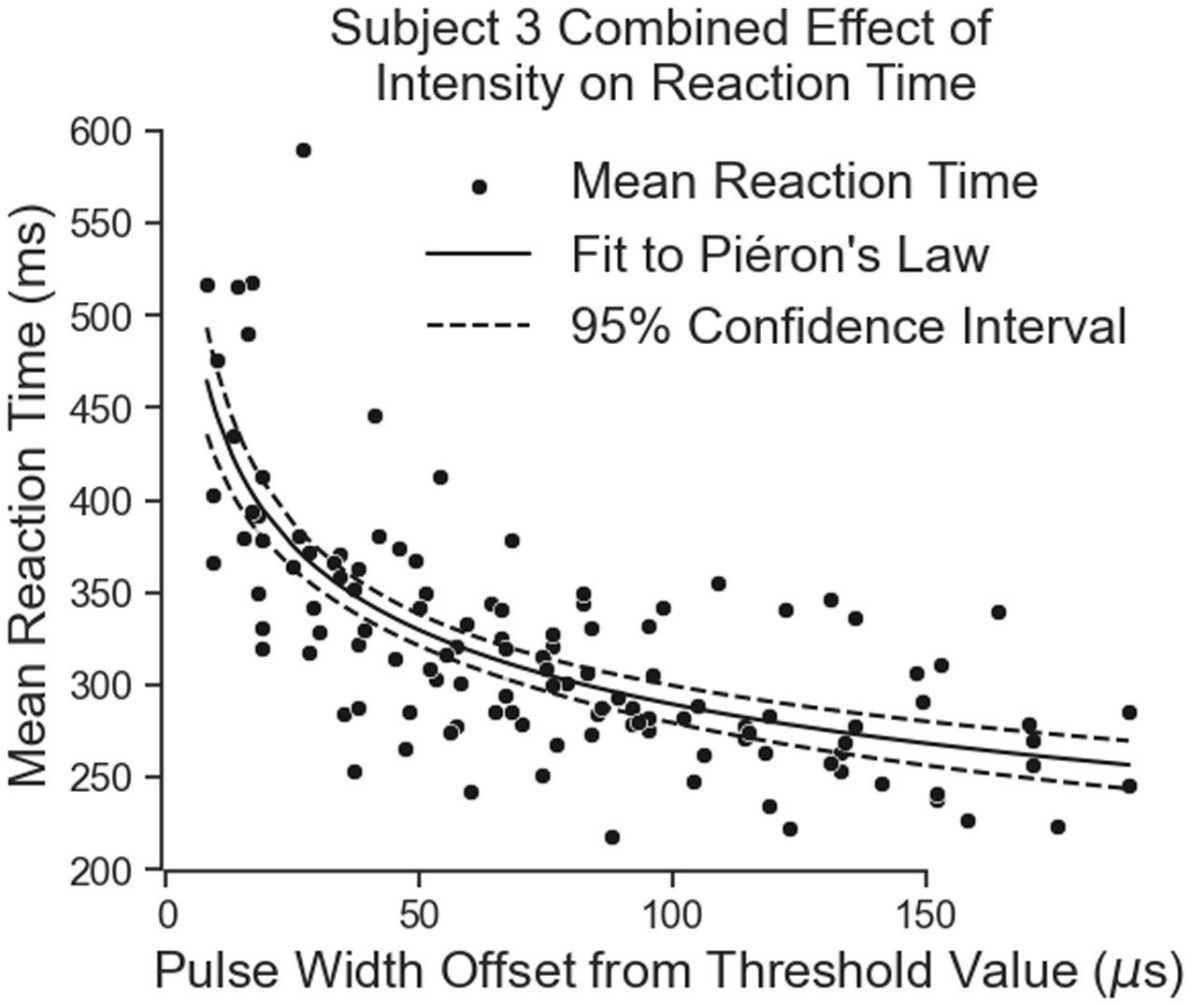
The combination of all of Subject 3’s data together follows Pieron’s Law for the intensity to stimulus processing time.

Both equations show similar fit parameters to Subject 1’s data as well as to each other, but the fits are much closer for Subject 3 compared to Subject 1. This is because Subject 3 was able to come in for 6 days of experiments while Subject 1 came for 3 days. When Subject 3’s data of both locations are combined and the curve fit redone, the variables are once again very similar to all the previous results (Figure 10; Table 3). The fit equation has a β value of 685.5 and an α value of 0.1871 with a root mean squared error of 45.27 ms, which is again similar to the individual fit errors.

Of note here is how similar Subject 1’s data is to Subject 3 despite having such a slow time to process peripheral nerve stimulation in the previous experiment. This may imply that removing the visual component of the test the subject commented as confusing him may have helped him “understand” the stimulus better.

These results suggest the effect of peripheral nerve stimulation intensity may be location independent, at least for these two subjects. Overall, the result of this test indicates that the brain responds to changes in peripheral nerve stimulation intensity in a similar way to naturally physical tactile feedback. Unfortunately, we were not able to collect the intensity-dependent data for the vibrotactile stimuli or visual stimuli as would have been stronger for direct comparison.

### Measuring the pre-perceptual tactile connections to the motor plan

All three subjects react to a comfortable level stimulus, i.e., stronger, faster than a weak stimulus alone, but all three test cases have much longer reaction times than results with a single strong stimulus only (Table 2). We believe this is from the subjects’ focus on counting and reacting adding a small amount of cognitive load.

**Figure 11:**
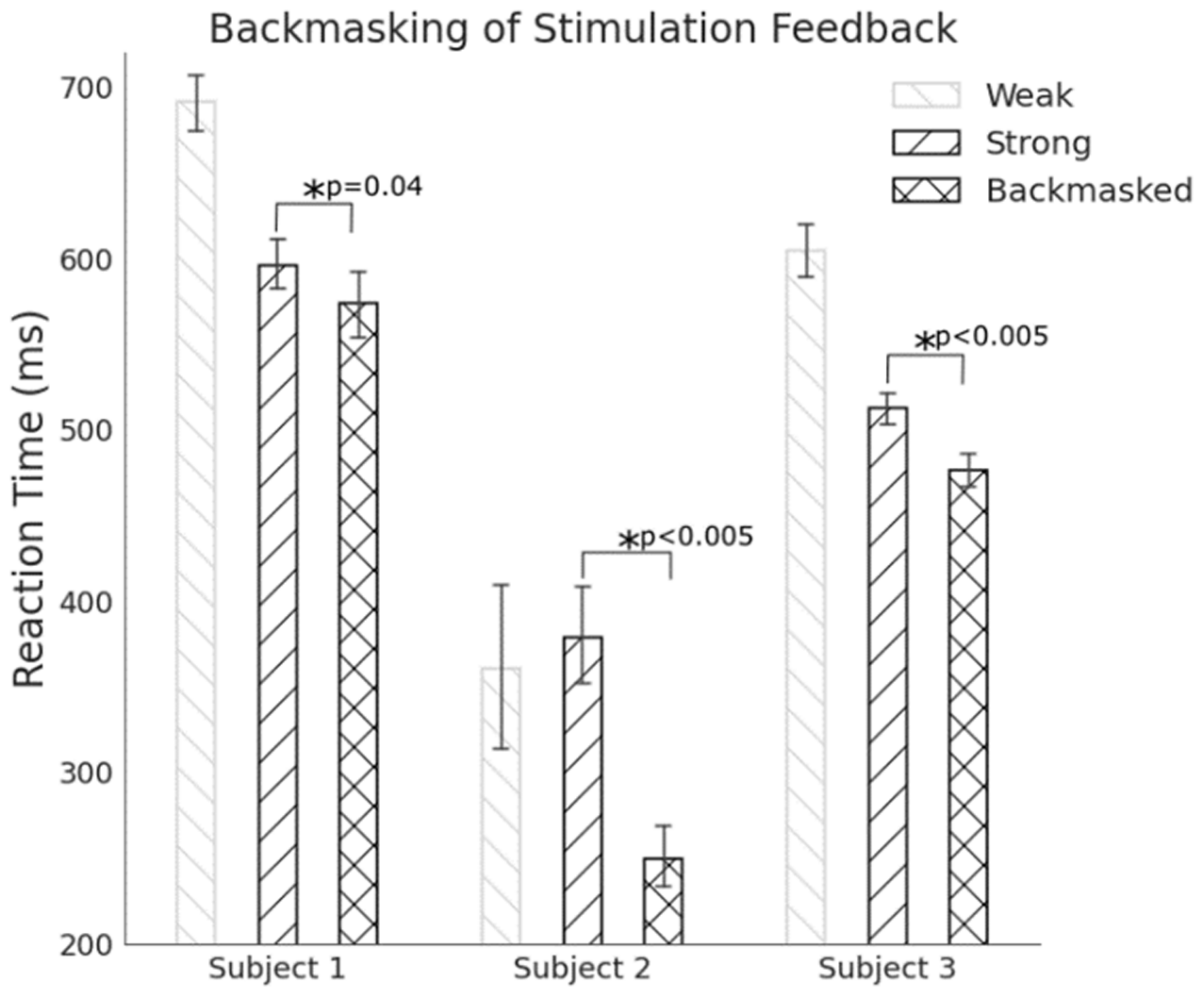
For all subjects, backmasking a tactile stimulus allowed for a faster reaction compared to when there was no masked stimulus indicating the pre-perceptual stimulus can prime a preplanned motor action.

All three subjects react faster to a masked stimulus compared to when there is no masked pre-perceptual stimulus (Figure 11). For Subjects 1 and 3, they participated in enough trials for their data to be distributed normally. Subject 2 only has about 50 trials and the data is not distributed normally. A non-parametric Mann–Whitney statistically tests the hypotheses for this subject. The central hypothesis is that a backmasked stimulus results in a faster reaction despite the subject only reporting the perception of a single pulse. During successful backmasking trials, the strong and backmasked cases are perceived identically by the subject Trials where the subject feel two pulses result in their exclusion. For all subjects there is a significant difference in their reaction to a perceived stimulus between the strong stimuli and the backmasked stimuli, supporting the hypothesis that the weak stimulus is priming them to execute their planned contraction earlier.

Subject 1 correctly classified the catch trials as two pulses 95.4% of the time, Subject 2 correctly classified the catch trials as two pulses 40.7% of the time, and Subject 3 correctly classified the catch trials as two pulses 98.7% of the time. Subject 2 had difficulties classifying the catch trials, and even the strong stimuli when presented alone. The subject would often state that the single pulses felt like two or even three pulses. Consequently, there are many fewer trials that qualify for analysis as backmasked trials in this subject compared to the others.

These results support the hypothesis that peripheral nerve feedback does not need to be perceived to interact with the motor system. This is known to occur with natural tactile feedback, but these results demonstrate that an unpatterned and artificially generated tactile feedback given through direct neural stimulation can interact with the nonconscious pathways of the brain in a similar way.

## Discussion

These three experiments demonstrate peripheral nerve stimulation interacts with the central nervous system pathways similar to natural tactile feedback. Throughout the study, simple reaction times tested three important aspects of peripheral nerve stimulation: the basic application of its feedback in triggering a motor action; the brain processes different reaction times related to different intensities of peripheral nerve stimulation; and peripheral nerve stimulation engages pre-perceptual tactile pathways to trigger a planned motor action.

The first experiment compared visual feedback to tactile feedback of either skin vibration or peripheral nerve stimulation. Artificial, non-patterned peripheral nerve feedback is processed at a speed faster than visual feedback alone. For Subjects 2 and 3, processing occurs at a speed similar to natural tactile feedback from vibratory feedback on the forearm. In Subject 1, however, peripheral nerve stimulation was statistically faster than vision, but slower than vibratory feedback. Subject 1 stated during the test that feeling a tactile sensation without seeing its source was even distracting to them. This was not the case with the vibration motor, as the subject could see and feel it touching his skin in between trials. Looking at this same subject’s intensity based reaction time data, his reaction times to the same intensity stimulus, shown by the right most points on Figure 7A, are faster and more comparable to the other subjects. The main difference in this test was Subject 1 closed his eyes during the test to exclude all visual distractions.

The combination of visual feedback and peripheral nerve stimulation also resulted in faster reaction times compared to visual feedback alone. We expected this difference to be on the order of 30–50 ms because visual feedback transduction in the retina occurs in about 50 ms with a short path to the brain and tactile transduction in hand occurs in about 4 ms with about a 20 ms travel time up the arm to the brain (Scott, 2016; Sombeck and Miller, 2019; Suresh et al., 2021). We were surprised to see a difference much greater of tactile feedback being processed up to 150 ms faster than visual feedback. This is possibly explained by Hansen who found that divided attention between the senses slows visual and auditory feedback, but not tactile feedback. They postulate that tactile feedback seems to need a more immediate response compared to all of the senses due to it being generated by something physically touching the body and thus is processed at a basic level at a pre-perceptual level (Scott, 2016). This widening of the gap between tactile and visual reaction times is further evidence that the artificial tactile feedback be provided in our tests is also processed at a pre-perceptual level. For all subjects, visual-tactile feedback from vibration or peripheral nerve stimuli resulted in similar reaction times. This either indicates that the multisensory integration of naturally generated visual feedback and artificial peripheral nerve stimulation is processed similarly to naturally generated visual tactile feedback or that one sensation dominated in the combination. Based on the work of Ernst we know that more relevant or precise information about a task tends to dominate and that sensations need to be collocated to be optimally integrated (Ernst and Bülthoff, 2004). Therefore, what we see here is less likely to be multisensory integration and more likely to be a “winner take all” with either tactile modality being the more relevant sensation. While our study likely did not achieve multisensory integration, Risso has shown that spatially locating peripheral nerve stimulation with visual feedback reduces processing times of the combined feedback. This shows that peripheral nerve stimulation can optimally integrate with the other senses (Risso et al., 2019).

While peripheral nerve stimulation is processed in a similar time to vibrotactile feedback in these simple tasks, the peripheral nerve stimulation results in a much more functionally relevant location of sensation on the subjects’ phantom hands. At this very low level of a task, the nuance of where the touch is felt is less important, especially when the subject knows how to use the incoming stimulus regardless of what it feels like (Williams et al., 2016). Using a vibration versus a stimulus felt on the hand is less relevant than the fact that the subject felt a touch. In more complex tasks like grasping and manipulating objects, this physiological mapping of perception to the hand is expected to become more relevant (Williams et al., 2016). If feeling the sensation on the hand provides quicker processing time, it is reasonable to hypothesize in future studies that it will also result in faster error and perturbation corrections. This concept was demonstrated for lower limb amputees in controlling their stance and walking given peripheral nerve stimulation versus vibration on the foot. Given tactile feedback felt on the foot, stimulation being on the phantom limb and vibration being on the intact limb. Either stimulus resulted in stance favoring the side of tactile feedback and there were no differences seen between the stimulation and the vibratory feedback *given that it is felt in a logical location on the foot* (Shell et al., 2021).

The second test investigated the change in reaction time due to changes in stimulation intensity. We expected that higher intensities of stimulation would reduce the time to react to feedback, but the trend of intensity-based reaction speed improvements to artificial stimulation had not been systematically demonstrated. Piéron’s Law describes this trend for many forms of natural sensations (Pins and Bonnet, 1996; Imanaka et al., 2002; Van Maanen et al., 2012; Loutit and Potas, 2020). If peripheral nerve stimulation has a similar intensity effect on simple reaction times to natural tactile feedback, we would also expect it to follow Piéron’s Law. The results of this experiment match Piéron’s Law describing the effect of different peripheral nerve stimulation intensities on the mean simple reaction time. Note that in this test, intensity of the stimulus was increased by modulating the pulse width of the stimulus, the location size of the stimulus also increased. Previous studies that using nylon fibers of different stiffnesses and widths showed a reduction of reaction times to touch followed Piéron’s law due to changing either stimulus variable independently (Lele et al., 1954). In our case, using the activation charge rate equation combines the stiffness factor and the location factor together. Stiffness is accounted for by the frequency of the stimulus as the frequency of stimulus changes the firing rate of a specific location in the same way a specific fiber diameter would increase the firing rate of an area with a stiffer fiber (Handler and Ginty, 2021). The location size change is accounted for by the pulse width and pulse amplitude terms of the activation charge rate equation as the higher either of these terms are, the larger the location is (Graczyk et al., 2016). Using activation charge rate as a quantitative metric of intensity shows that the effect of modulating peripheral nerve stimuli followed the expected trend for modulating force and size of naturally generated tactile feedback.

Finally, when peripheral nerve stimulation supplies feedback masked from perception, it can still interact with the motor system by triggering a preplanned motor output. A backmasking trial created pre-perceptual feedback by masking a weak stimulus with a delayed strong stimulus. The masked, weak stimulus resulted in a faster reaction time to the strong pulse even though the subjects’ perception of the paradigms were the same. This supports the hypothesis that artificial tactile feedback is processed by pre-perceptual pathways of the neuroaxis, even though the artificial stimulation does not activate the sensory neural population exactly as would be expected from physical tactile feedback to the hand. While the triggered motor plan was faster given a masked stimulus, all reactions to the strong stimulus were slower than we expected given the results to our simple reaction time test and intensity-based reaction time tests. Based on our earlier results, we expected reaction times to comparable stimuli intensities in the range of 200 ms–300 ms. Instead, we saw reaction times closer to 500 ms–600 ms. We believe this delay in reaction is due to the subjects’ focus being split between two tasks: reacting and verbally stating the number of pulses they felt. This addition of tasks may increase (Hanson et al., 2009) the number of processing steps between perceiving a stimulus and reacting to it. For example, Miller defines the steps to reacting to a simple reaction time as stimulus detection and motor execution (Miller et al., 1999; Miller and Low, 2001). This is what occurred in the first two tests of this study. Adding a discrimination step to the reaction, for example deciding to react or not to two different stimuli adds one more step and adding a choice of how to react adds another (Miller et al., 1999; Miller and Low, 2001). Adding a step to state the number of pulses felt likely added a step for discriminating the stimulus felt. In addition to this, the verbal system is not as closely connected to the tactile and motor systems as the tactile and motor systems are connected to each other. In tests where tactile stimuli were given to individual who could not perceive tactile stimuli, they were able to instinctively move their hand in response but were unable to respond verbally as to how they were touched (Dijkerman and Haan, 2007). Adding a verbal response to the stimulus discrimination task likely further slowed the time to react.

Our backmasking test results align well with other studies using natural tactile feedback in backmasking reaction time tests (Imanaka et al., 2002) and suggest the stimulation interacts with some per-perceptual pathway of the central nervous system but does not directly identify the specific pre- pathways artificial stimuli use. These pathways include both subcortical regions and cortical pathways that do not evoke perception. We do know that at least one of these pathways must have been used for an unperceived stimulus to trigger a motor plan, but imaging of activity in the brain would be needed to see which pre-perceptual pathways activate.

None of the tests in this study involved matching tests on the intact hand of the subjects. This was for two main reasons. The first is that the path length of the nerves to the neuroaxis affects the time to react to stimuli. This is why the vibration motors in the simple reaction tests were placed above the location of the nerve cuffs in each subject. Secondly, the two sides of the body react with different speeds based on which side was the dominant one for the subject (Dexheimer et al., 2022).

These studies strongly support the hypothesis that peripheral nerve stimulation processing by the ascending tactile pathway is similar to natural tactile feedback. Artificial tactile feedback results in significantly faster reaction than visual feedback. This indicates that peripheral nerve stimulation provides functional benefit beyond just the perception of touch to upper limb loss subjects that currently rely exclusively on visual feedback in functional tasks with the prosthesis. Peripheral nerve stimulation reaction time was similar to vibrotactile feedback, Further, reaction times followed an expected intensity-dependent trend as natural sensation. Simple, constant pulse intensity artificial stimulation engages fundamental processing pathways of the brain to refine usage in the higher levels of the brain. Finally, peripheral nerve stimulation interacts with the motor system without the need for tactile perception.

The results of this study demonstrate different timing response than those reported for stimulation the primary somatosensory cortex using cortical arrays. First, the cortex is typically engaged for complex or novel tasks requiring thought and planning. This is evident from the fact that as new tasks are presented to individuals, large areas of the cortex show activity, but as tasks become more routine, very small areas show activity as less conscious effort is needed (Dehaene and Changeux, 2011). Therefore, tests of cortical stimulation that measure simple reactions and preplanned actions are not the natural pathways for those tasks and would be expected to have reaction times that do not necessarily match reaction time from physical feedback. Simple reaction times and small motor corrections are usually first processed in the brain stem which is more naturally engaged through the dorsal column nuclei along the medial lemniscus pathway to the somatosensory cortex. In fact, recent studies of the dorsal column show that its nuclei output signals more similar to the somatosensory cortex outputs than to the peripheral input. Therefore, the dorsal column nuclei are performing meaningful processes on the peripheral inputs before moving along the medial lemniscus pathway to the cortex (Suresh et al., 2021).

Peripheral nerve stimuli appear to add benefit beyond simple “feeling of touch” but also for pre-perceptual processing of tactile information lost in those missing a limb. Peripheral feedback providing this “hidden” information is true for most people excluding those with high spinal cord injuries resulting in complete loss of sensation. For these individuals, stimuli in the periphery are not feasible. Based on the results of this study, it would suggest that stimuli just above the level of injury in the spine would benefit from the processing in the brain stem. Alternatively, stimuli in the cortical areas that the brain stem nuclei lead to may also improve processing of feedback given near the end of the tactile pathway. Further studies need to be done into the areas that natural touch goes to simultaneously and how the whole brain shifts in its processing of tactile feedback as tasks become more routine. Targeting the right areas during the learning of a motor task may help push a learned sensorimotor task into a more pre-perceptual, routine version.

Peripheral nerve stimulation integrates well with the low-level perceptual structures of the brain, but little is known in how it will combine with the motor system is more complex processing and utilization. Mainly, how is peripheral nerve stimulation used in error correction? Quick and intuitive corrections to errors during tasks would imply two important points. Firstly, peripheral nerve stimulation would be useful outside of the highly controlled nature of the lab in real world tasks. Secondly, quick error corrections, especially those that are faster than the simple reaction times found in this study, would indicate subcortical usage or even reflexive usage likely by the cerebellum. While this study did not find the specific pathways that peripheral nerve stimulation took advantage of, there are multiple methods that would elucidate this. Two of the main regions of the brain that alter the motor plan at a pre-perceptual level are the cerebellum and the posterior parietal cortex. Both of these areas can also be disrupted by transcranial magnetic stimulation (TMS). If TMS over either of those areas eliminates pre-perceptual usage in a backmasking test, it would indicate if the brain stem path or the pre-perceptual cortical path was more important.

This work presents an important first step in investigating the integration of artificial tactile feedback in lower levels of sensorimotor integration. With the information in this study, we have shown that even a stimulus with little to no information is still has enough information content to integrate with the lowest levels of the motor plan.

## Data availability statement

The raw data supporting the conclusions of this article will be made available by the authors, without undue reservation.

## Ethics statement

The studies involving humans were approved by Louis Stokes Cleveland VA Medical Center IRB. The studies were conducted in accordance with the local legislation and institutional requirements. The participants provided their written informed consent to participate in this study.

## Author contributions

NC: Conceptualization, Data curation, Formal analysis, Investigation, Methodology, Software, Visualization, Writing – original draft, Writing – review & editing. DT: Funding acquisition, Project administration, Resources, Supervision, Writing – review & editing.
