# Supplementary figures and images for: Utilization of peripheral nerve feedback at a preconscious level
Source: Front Neurosci. 2024 Mar 14;18:1336431. doi: 10.3389/fnins.2024.1336431 (PMC10977079; doi:10.3389/fnins.2024.1336431)

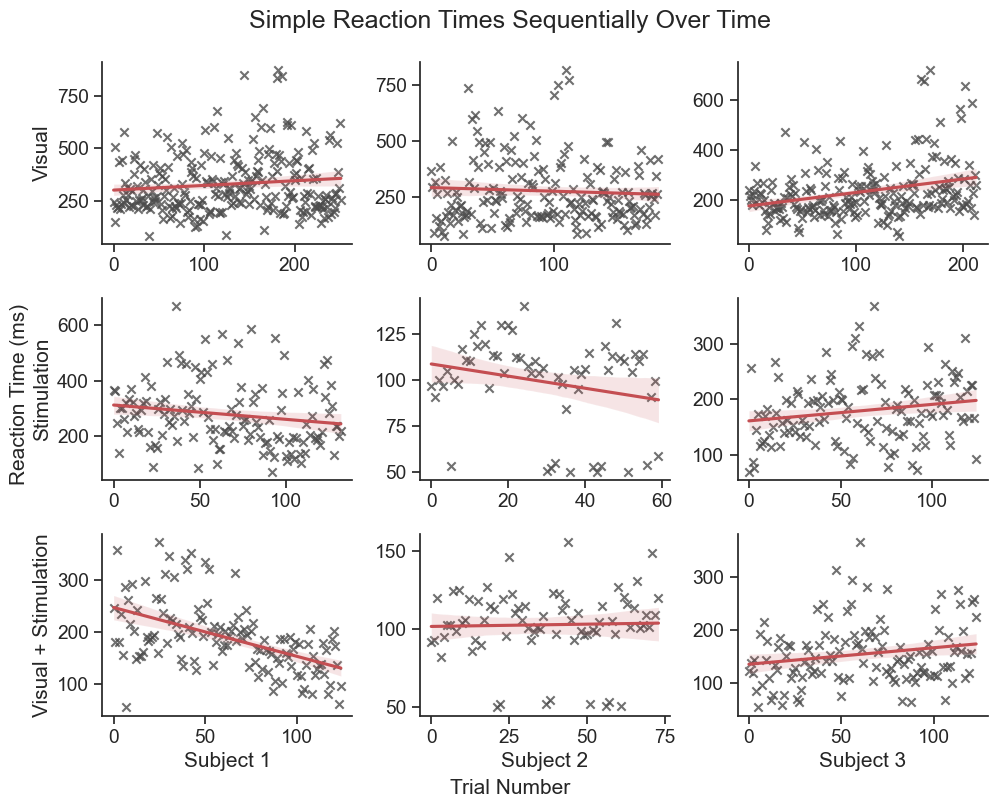

Supplement: Supplementary file 1 [file Image_1.TIFF]
